# piR-121380 Is Involved in Cryo-Capacitation and Regulates Post-Thawed Boar Sperm Quality Through Phosphorylation of ERK2 *via* Targeting PTPN7

**DOI:** 10.3389/fcell.2021.792994

**Published:** 2022-01-26

**Authors:** Yihan Wang, Xiang Yuan, Malik Ahsan Ali, Ziyue Qin, Yan Zhang, Changjun Zeng

**Affiliations:** ^1^ Farm Animal Genetic Resources Exploration and Innovation Key Laboratory of Sichuan Province, College of Animal Science and Technology, Sichuan Agricultural University, Chengdu, China; ^2^ Department of Theriogenology, Riphah College of Veterinary Sciences, Lahore, Pakistan

**Keywords:** boar sperm, piR-121380, PTPN7, ERK1/2 phosphorylation, cryo-capacitation

## Abstract

Cryopreservation induces capacitation-like (cryo-capacitation) changes, similar to natural capacitation, and affects the fertility potential of post-thawed sperm. The molecular mechanism of sperm cryo-capacitation during cryopreservation remains unknown. PIWI-interacting RNAs (piRNAs) have been reported to be involved in cryo-capacitation of post-thawed sperm and regulation of sperm motility, capacitation, and chemotaxis. In this study, protein tyrosine phosphatase nonreceptor type 7 (PTPN7) was positively targeted by piR-121380 after a dual luciferase assay. The mRNA expression of PTPN7 and piR-121380 was significantly decreased (*p* < 0.01); however, PTPN7 protein was significantly increased (*p* < 0.01) in post-thawed boar sperm. Furthermore, E1RK1/2 phosphorylation was reduced during cryopreservation. Six hours after transfection with piR-121380 mimic and inhibitor, the phosphorylation of ERK2 was significantly increased and decreased (*p* < 0.01), respectively. Furthermore, the highest and lowest total sperm motility, forward motility, and capacitation rate were observed after piR-121380 mimic and inhibitor treatments, respectively. The concentration of intracellular calcium ([Ca2+]i) showed no significant difference after transfection with either piR-121380 mimic or inhibitor at 1, 3, and 6 h. In conclusion, we demonstrated that piR-121380 modulates ERK2 phosphorylation by targeting PTPN7, which induces sperm cryo-capacitation, and eventually affects the motility and fertility potential of post-thawed sperm.

## Introduction

Sperm cryopreservation is an invaluable technique involved in artificial insemination (AI) and the most efficient way to preserve male fertility (Yeste et al., 2017). However, the structural and functional aspects of sperm are strongly damaged during cryopreservation, such as chromatin integrity, sperm viability, motility, mitochondrial and plasma membranes, and acrosome integrity, resulting in decreased fertilizing ability (Ozkavukcu et al., 2008; Vadnais and Althouse, 2011; Yeste et al., 2015; Yeste, 2016). In addition, the freezing–thawing procedure also induces capacitation-like changes, including cholesterol efflux, influx of HCO_3_(−), increased intracellular Ca^2+^, activation of cAMP/PKA signaling pathway, and protein tyrosine phosphorylation (Cerolini et al., 2000; Xia and Ren, 2009; Kumar and Atreja, 2012; Treulen et al., 2018), similar to natural capacitation. However, these similar changes are not completely analogous and should not be classified as true capacitation (Green and Watson, 2001).

Sperm undergoes physiological and biochemical changes and then acquires fertilizing ability after capacitation (Brener et al., 2003; Grasa et al., 2006; Visconti et al., 2011). Protein tyrosine phosphorylation is involved in epididymal sperm maturation, capacitation, acrosomal reaction, and motility (Roberts et al., 2003; Luconi et al., 2005; Mor et al., 2007). Additionally, tyrosine phosphorylation of acrosin-binding protein (ACRBP), ERP99, HSP60, as well as sperm head proteins facilitates the binding of sperm to the zona pellucida in boar sperm (Asquith et al., 2004; Kato et al., 2021). Compared with natural capacitation, cryo-capacitation-induced protein tyrosine phosphorylation is involved in decreased sperm-binding to zona pellucida across various species (Cormier and Bailey, 2003; Kadirvel et al., 2011). Moreover, different freezing protocols result in different levels of protein tyrosine phosphorylation (Kumaresan et al., 2012). Freezing extenders containing egg yolk have been reported to produce a 33-kDa tyrosine-phosphorylated protein in frozen–thawed boar sperm (Orrego et al., 2019).

The level of protein tyrosine phosphorylation is strictly regulated by downstream protein tyrosine kinases (PTKs) and protein tyrosine phosphatases (PTPs), which links the cAMP pathway that affects protein tyrosine phosphorylation. Additionally, PTKs and PTPs are essential for regulation of human sperm acrosome reaction (Tomes et al., 2004). Seligman et al. observed that although PTP activity was decreased, PTKs remained active, and protein tyrosine phosphorylation was increased in the sperm tail from caput of epididymis (Seligman et al., 2004). Previous studies reported that extracellular signal-regulated kinase1/2 (ERK1/2), a class I PTP cluster (DSP), also play an important role in promoting sperm hyperactivated motility and acrosomal reaction (Almog et al., 2008; Li et al., 2009). However, the expression and function of PTP during sperm cryopreservation remain unknown.

It is well known that PIWI-interacting RNAs (piRNAs) are widely involved in physiological processes including gametes development, germ stem cell differentiation, embryonic development, and sex determination (Malone and Hannon, 2009; Kamminga et al., 2010; Zhao et al., 2013; Kiuchi et al., 2014; Ma et al., 2017; Halbach et al., 2020). Our previous study showed that piR-121380 was differentially expressed between fresh and post-thawed boar sperm. It was speculated to target PTPN7 and be involved in olfactory transduction pathways, which were thought to be involved in sperm motility, acrosome reaction, and sperm-egg recognition (Fukuda et al., 2004; Spehr et al., 2004; Milardi et al., 2017). In this study, we demonstrated that piR-121380 modifies ERK2 phosphorylation by targeting PTPN7, induces sperm capacitation-like changes and eventually affects the motility and fertility potential of post-thawed sperm.

## Materials and Methods

### Ethical Statement

All procedures starting from semen collection to treatments were implemented strictly considering the Regulation of the Administration of Affairs Concerning Experimental Animals (Ministry of Science and Technology, China, revised in June 2004) approved by the Institutional Animal Care and Use Committee in the College of Animal Science and Technology, Sichuan Agricultural University, Sichuan, China, under permit No: 2019202012.

### Semen Collection and Treatment

Fresh ejaculates (*n* = 10) were collected by glove-handed technique from healthy and mature Yorkshire boars. Immediately, the fresh semen was diluted with semen extender (Zenoaq, Zenolong, China) in the ratio of 1:3. After stirring the mixture, the semen was placed in a temperature-controlled container (17°C) and transported to the laboratory. All the fresh ejaculates were confirmed to exhibit normal morphology, more than 80% viability, 90% motility, and over 1 × 10^8^ ml^−1^ density for subsequent experiments.

Then, all the fresh samples were mixed and equally divided into three aliquots: (1) The first aliquot was directly frozen in a liquid nitrogen (LN) tank and then stored at −80°C for RNA and protein extraction; (2) the second aliquot was cryopreserved as described in the next section; (3) the third aliquot was divided into two groups: (1) negative control group (NC) and (2) capacitation group (capa-). The negative control group was centrifuged (1,500 r/min, 5 min) and then incubated in BTS medium (37 g glucose, 3 g trisodium citrate, 1.25 g Na_2_-EDTA, 1.25 g NaHCO_3_, 0.75 g KCl, 0.6 g/L penicillin G sodium, and 1.0 g/L dihydrostreptomycin; all diluted to 1 L) for 1, 3, and 6 h at 37°C. The capacitation group was incubated with 40 ng/ml heparin as described previously (Parrish et al., 1985). Notably, fresh semen collected from pig farm was centrifuged and then diluted with suitable BTS to prepare the electro-transfection group for subsequent determination of parameters, RT-PCR, and Western blot. The following experiments with frozen–thawed group, capacitation group, and transfection group were independent.

### Semen Cryopreservation

The second aliquot was cryopreserved according to our laboratory’s procedure (Zeng et al., 2014; Ran et al., 2019). Firstly, semen was centrifuged at 1,500 r/min for 5 min (17°C) to discard the seminal plasma and semen extender. Secondly, lactose-egg yolk (LEY) extender I (11% β-lactose, 20% hen’s egg yolk) was added to sperm suspension and mixed slowly. This compound (containing extender I and sperm) and extender II (LEY extender was supplemented with the same volume of 6% glycerol to yield a final concentration of 3% glycerol) were cooled slowly to 4°C within 2 h. After equilibrating for 2 h, the compound was mixed with the same volume of extender II. Finally, the miscible liquids were inhaled into 0.25-ml straws (FHK, Tokyo, Japan) and equilibrated approximately 3 cm above LN vapors (∼130°C) for 10 min and then immersed into LN (−196°C). Thawing was performed at 37°C for 30 s and then transferred to BTS (37°C).

### RNA Stability Assay

Fresh and frozen–thawed sperm were incubated with 5 μg/ml actinomycin D to perform mRNA stability assay as described previously (Rodrigues et al., 2009), and RNA was extracted at time points of 0, 2, 4, 6 and 8 h (Chen et al., 2008). The PTPN7 level was measured by qPCR as described previously. 18S rRNA gene was used as an endogenous control for mRNA normalization in the mRNA stability assay. The mRNA decay was determined by non-linear regression curve fitting (one phase decay) using GraphPad Prism (v.8.0). The parameters were set as follows: least squares (ordinary fit), confidence level (95%), asymmetrical (likelihood) CI, goodness of fit by R square, and convergence criteria (medium).

### Sperm Electro-Transfection

Electro-transfection was performed based on a previously standardized method (Wang et al., 2020). Initially, fresh ejaculates were centrifuged (1,500 r/min, 5 min, 17°C) to discard the supernatant and then diluted with suitable BTS to yield a final concentration of 4 × 10^7^ ml^−1^. Then, transfection was conducted by Cell Manipulation ECM-2001 (BTX, Holliston, MA, United States) with 20 nM of piR-121380 mimic, inhibitor, mimic NC, inhibitor NC, and negative control (NC) (Ribobio, Guangzhou, China); pulse conditions were adjusted as 4 × 300 V for 100 µs. The mixture was incubated at 37°C within 12 h, and after 12 h at 17°C after transfection. The relative expression of piR-121380 and PTPN7 was assessed at 17°C from 12 to 60 h. Western blot was performed at 1, 3, and 6 h after transfection. Similarly, capacitation rate and intracellular calcium were determined at 1, 3, and 6 h after transfection (37°C).

### RNA Extraction, cDNA Synthesis, and Quantitative Real-Time PCR

Total RNA of all ejaculates was extracted using a Trizol LS Reagent kit (Invitrogen, Carlsbad, CA, United States) as described previously (Ran et al., 2019). RNA with the optimal OD260/280 of 1.8–2.0 was selected for the subsequent reverse transcription. The total RNA was reversed by HiScript III RT SuperMix for qPCR Kit (Vazyme, Nanjing, China) and Takara SYBR PrimeScript miRNA RT-PCR Reagent Kit (Takara Biotech, Dalian, China), respectively, according to the manufacturers’ instructions. qPCR was performed on the CFX 96 Real-Time PCR Detection System (Bio-Rad, Hercules, CA, United States). Three biological replicates were set for each group, and the results were accounted for using the 2^−∆∆CT^ method (Livak and Schmittgen, 2001). Internal controls: 18S rRNA, GAPDH gene, and U6 (for piRNA) were used as the reference for normalization in relative mRNA and piRNA expression analysis (Table 1).

### Target Prediction of piR-121380 and Dual Luciferase Reporter Assay

Potential target genes of key piRNAs were predicted by miRanda (John et al., 2004), and the binding site was verified using R adhering to the principle as previously described (Zhang et al., 2015). The wild-type vector (pWT-PTPN7) was designed to include the piR-121380 binding site, 200 base pairs upstream and downstream. The mutant type vector was designed by mutating a few bases of the binding site. The wild-type and mutant vectors were amplified and inserted into pmirGLO Luciferase reporter vector (Tsingke, Nanjing, China) to construct the wild-type and mutant plasmids.

Hela cells were cultured in 24-well plates at 10^5^ cells/well in Dulbecco’s modified Eagle’s medium (DMEM) containing 10% fetal bovine serum (FBS) at 37°C in a humid CO_2_ incubator (5% CO_2_). Transfection was performed until cell confluency was above 70%. The transfection groups were prepared as follows: pWT-PTPN7-mimic, pWT-PTPN7-mimic NC, pMT-PTPN7-mimic, and pMT-PTPN7-mimic NC, which were individually co-transfected into Hela cells using FuGENE HD Transfection Reagent (Promega, Madison, WI, United States). After 6 h, luciferase activity was measured with the Dual-Luciferase Reporter Assay System (Promega, Madison, WI, United States) according to the manufacturer’s instructions. The final luciferase activity was normalized with Renilla luciferase activity.

### Sperm Motility Detection

Fresh ejaculates, after transfection, were subjected to 12–60 h of incubation under liquid preservation conditions. Prior to measuring sperm motility, the sperm counting chamber (Leja®, Holland) was pre-heated on 37°C hot-stage and the CASA detection system (Hamilton, Germany) was debugged until the camera image was clear (Dardmeh et al., 2021). The following calibration parameters were considered for this study: immotile is defined by lateral head displacement (ALH) < 1 µm and curvilinear velocity (VCL) < 24 μm/s; total motility is defined by ALH > 1 µm and VCL > 24 μm/s; progressive motility is defined by straight-line velocity (VSL) > 10 μm/s and VCL > 48 μm/s; non-progressive motility is defined by VSL < 10 μm/s and 24 < VCL< 48 μm/s. Five microliters of semen was dropped on the pre-heated slide after maintenance for 15 min in a water bath and tested for sperm motility. Five visual fields containing more than 200 sperm were selected to measure the sperm parameters. All the procedures were repeated at least three times to minimize errors.

### Assessment of Acrosomal Status and Capacitation

After transfection for 1, 3, and 6 h, sperm was washed and then fixed with paraformaldehyde for 10 min. Peanut agglutinin (PNA) was used to measure the acrosomal status according to the method used by Mendoza et al. (1992). Sperm was cultured with FITC-PNA (Sigma-Aldrich, United States) working fluid (20 μg/ml) at 37°C for 20 min and then 1 µl of PI (Sigma-Aldrich, United States) solution was added into the mixture and co-incubated at 37°C for 5 min. The samples were washed three times with PBS. Epifluorescence microscopy (Olympus, Tokyo, Japan) was used to evaluate the acrosomal status of sperm with no less than 200 sperm per replicate and ImageJ was used to overlap the red and green fluorescence.

### Measurement of Intracellular Calcium

The concentration of intracellular calcium was measured with Fluo 4, AM (Yesean, Shanghai, China). After transfection and incubation with 40 ng/ml heparin, sperm were washed once and re-suspended in HBSS buffer with 4 µM Fluo 4, AM (Solarbio, Beijing, China). The sperm suspension was incubated at 37°C for 30 min and then washed three times. After that, another incubation was performed at 37°C for 20 min to ensure complete de-esterification of AM groups in sperm. The fluorescence of sperm was detected using a microplate reader (Thermo Scientific, United States) at 494 nm excitation/516 nm emission. The [Ca^2+^]_i_ was determined by the following equation: ΔF/F0 (%) = (F − F0)/F0 × 100%. F indicates the fluorescence intensity of each group, and F0 indicates the fluorescence intensity of sperm suspension without treatment.

### Immunolocalization of ERK1/2 and PTPN7 in Boar Sperm

Indirect immunofluorescence was used to detect the localization of PTPN7, ERK1, and ERK2 protein in boar sperm. Sperm precipitate was fixed with 4% paraformaldehyde for 10 min, washed with PBS, and permeabilized with 0.5% Triton X-100 (Beyotime Biotechnology, Shanghai, China). Then, 5% bovine serum albumin (BSA) (Sigma-Aldrich, United States) was added and incubated for 30 min. After that, the samples were incubated with primary antibodies including anti-PTPN7 (Origene, Beijing, China), ERK1 (Proteintech, 11257-1-AP), and ERK2 (Proteintech, 16443-1-AP) polyclonal antibodies diluted 1:25 in Western primary anti diluent (Beyotime Biotechnology, Shanghai, China) at 37°C for 1 h. After three washes, the samples were incubated with secondary antibody of goat anti-rabbit IgG (H + L) conjugated with CoraLite 594 (Proteintech, SA00013-4) diluted at 1:25 in Western secondary anti diluent (Beyotime Biotechnology, Shanghai, China) at 37°C for 1 h. After three washes with PBST (PBS, 1% Tween, and 0.02 g glycine), sperm were observed and evaluated using a fluorescence microscope equipped with a DP70 camera (Olympus, Tokyo, Japan).

### Western Blotting Analysis

Total protein was extracted from sperm using RIPA lysis buffer (Beyotime Biotechnology, P0013B, China) supplemented with protease and phosphatase inhibitors, containing 1% PMSF (Beyotime Biotechnology, ST506, China), phosphatase inhibitor cocktail, and 0.05 M EGTA (Beyotime Biotechnology, P1045, China). The denatured proteins dissolved in SDS-PAGE sample loading buffer were separated by 10% Hepes gel (Beyotime Biotechnology, P0508S, China) and transferred to PVDF membrane (Beyotime Biotechnology, FFP32, China). The membrane was then blocked with blocking buffer (Beyotime Biotechnology, P0252, China) at room temperature for 1 h. Then, the membrane was incubated with anti-PTPN7 (1:500, Proteintech, 15286-1-AP, China), anti-ERK1/2 (1:500, Cell Signaling Technology, 4695, United States), anti-phospho- ERK1/2 (1:1,000, Cell Signaling Technology, 9101, United States), and anti-β-tubulin (1:1,000, Abcam, ab179513, United States) antibodies diluted in primary antibody diluent (Beyotime Biotechnology, Shanghai, China) overnight at 4°C. Notably, each incubation was an individual experiment. The membrane was then incubated with goat anti-rabbit IgG conjugated with HRP (1:4,000, Proteintech, SA00001-2, China) at room temperature for 1 h, and washed three times with PBST. Enhanced chemiluminescence (ECL) detection was then performed using BeyoEcl Moon kit (Beyotime Biotechnology, P0018FS, China) and Immun-StarTM Western CTM Chemiluminescence Kit (BIO-RAD, Hercules, CA, United States). The intensities of bands were quantified with ImageJ software. At least three independent experiments were performed.

### Statistical Analysis

The results are presented as mean ± standard error of mean (SEM). The differences between two groups were analyzed by independent sample *t*-test using SPSS (v.19.0) and R (v.4.0) in sperm viability, qPCR, and Western blot experiment (mimic versus mimic NC, inhibitor versus inhibitor NC). The differences among the groups were analyzed by one-way analysis of variance (ANOVA) followed by Dunnett post-test comparing each treatment column to control column. The relative piR121380 gene expression level was quantified by the 2^−∆∆CT^ method (Livak and Schmittgen, 2001). The gray values of protein bands were calculated using ImageJ (v. 1.48). To compare the effect of cryopreservation on the time–response curves of PTPN7 mRNA, nonlinear regression was performed using GraphPad Prism (v.8.0). *p* < 0.05 was considered statistically significant.

## Results

### PTPN7 is a Target Gene of piR-121380

**FIGURE 1:**
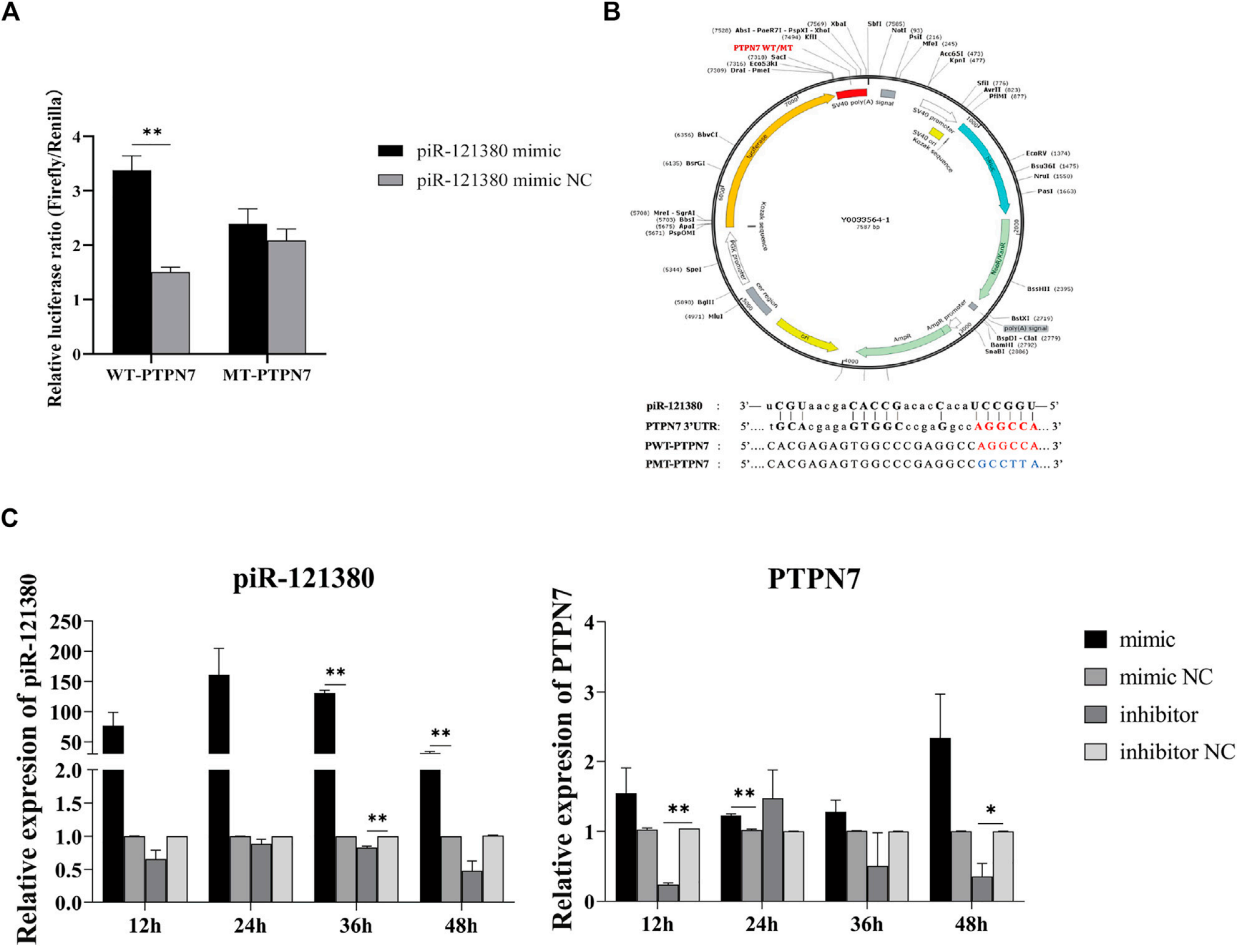
Target gene prediction of piR-121380 and dual-luciferase reporter assay. **(A)** The result of dual-luciferase reporter assay. **p < 0.01. **(B)** The structure of recombinant plasmid and the sequences of wild and mutant types and the predicted target site of piR-121380 on the 3′ UTR of PTPN7 in boar. Imperfect base pairing between piR-121380 and 3′UTR of PTPN7. **(C)** The expression of piR-121380 and PTPN7 after transfection from 12 to 48 h, respectively. *p < 0.05. **p < 0.01. The data are representative of at least three independent experiments (mean ± SEM).

Previous studies showed that the 3′ UTR of PTPN7 harbors only one potential target site for piR-121380 (Figure 1B), which is consistent with the preference of piRNA to the 3′UTR region of mRNA (Dai et al., 2019). The dual luciferase results showed a positive correlation between piR-121380 and PTPN7 expression (Figure 1A). The expression of piR-121380 was significantly increased (*p* < 0.01) in the pWT mimic group as compared to the mimic NC group, while no significant difference (*p* > 0.05) was observed between the pMT mimic group and the mimic NC group. To further explore the target relationship between piR-121380 and PTPN7, the expression of piR-121380 and PTPN7 was observed every 12 h up till 48 h after transfection. The results showed that the expression of piR-121380 increased from 12 to 48 h and significantly increased (*p* < 0.01) at 36 and 48 h in the mimic group as compared to the mimic NC group (Figure 1C). Similarly, the expression of PTPN7 decreased (*p* < 0.05) in the inhibitor group as compared to the inhibitor NC group from 12 to 48 h, except at 24 h (Figure 1C). Therefore, these findings demonstrated that PTPN7 is a target gene of piR121380, and piR-121380 has an effect on the expression of PTPN7 at the mRNA level.

**TABLE 1:** Primers used for quantitative reverse transcription PCR (RT-qPCR).

Gene	Sequence (5′-3′)	(^°^C)	Product size (bp)	(NCBI accession)
*GAPDH*	F: ACT​CAC​TCT​TCT​ACC​TTT​GAT​GT	60.0	100	XM_021091114.1
	R: TGT​TGC​TGT​AGC​CAA​ATT​CA			
*18S rRNA*	F: CCC​ACG​GAA​TCG​AGA​AAG​AG	60.0	132	Guo et al. (2018)
	R: TTGACGGAAGGGCACCA			
*PTPN7*	F: ATC​TTG​CCA​AAC​CCC​CAG​AG	60.0	119	XM_021064534.1
	R: GCG​ATG​TAG​GCC​TTG​TCC​TT			
*ERK1*	F: CAG​TCT​CTG​CCC​TCC​AAG​A	60.0	146	XM_013991188.1
	R: AGG​TAA​GGA​TGA​GCC​AGT​GC			
*ERK2*	F: CCC​CAT​CAC​AGG​AAG​ACC​T	57.8	121	NM_001198922.1
	R: GCT​TTG​GAG​TCA​GCA​TTT​GG			

### Effect of Cryopreservation and Capacitation on the Expression of piR-121380, PTPN7 and ERK1/2

**FIGURE 2:**
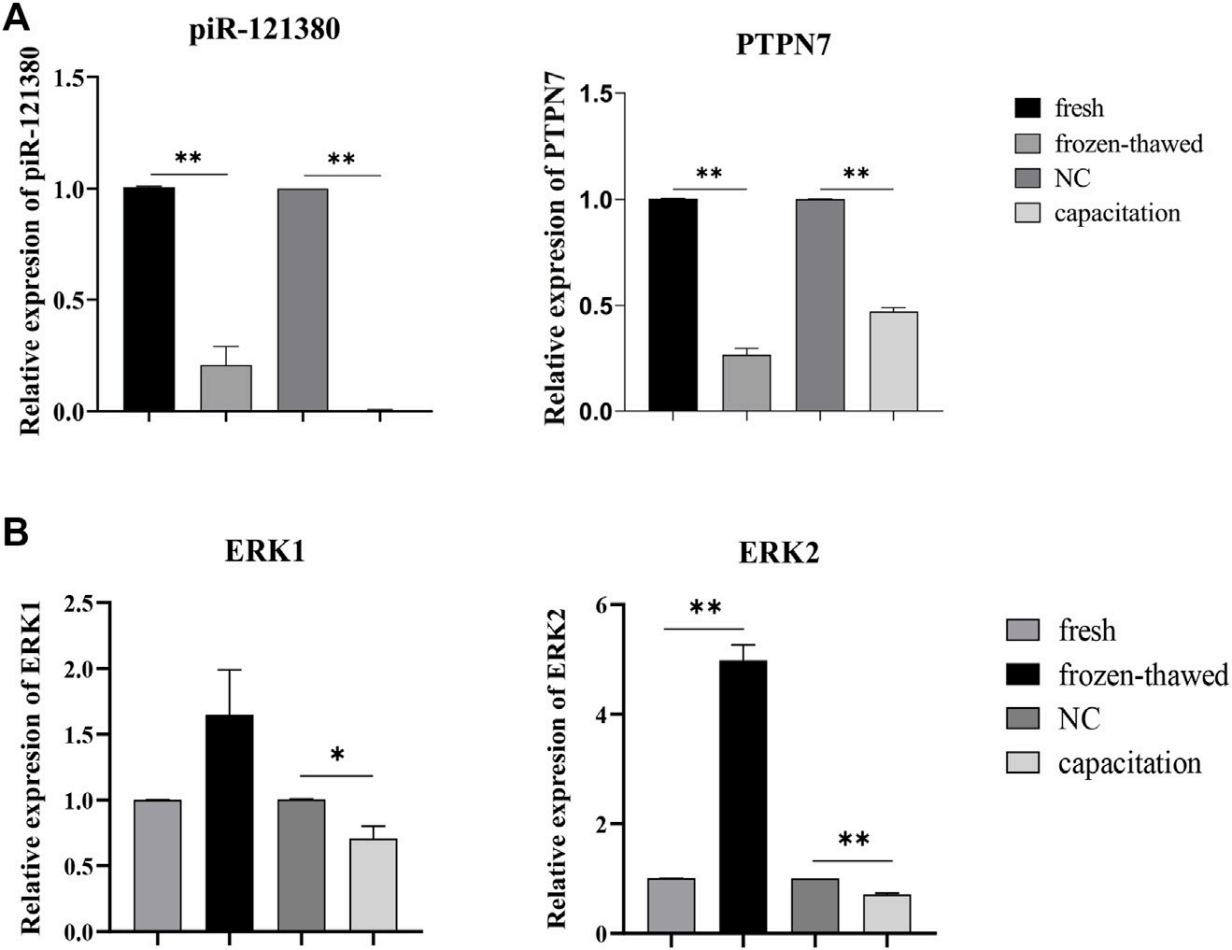
**(A)** The expression levels of piR-121380 and PTPN7 in fresh, frozen–thawed, and capacitated sperm. **p < 0.01. **(B)** The expression levels of ERK1 and ERK2 in fresh, frozen–thawed, and capacitated sperm. **p < 0.01. *p < 0.05. The data are representative of at least three independent experiments (mean ± SEM).

The RT-PCR results showed that the expression of piR-121380 and PTPN7 was significantly decreased (*p* < 0.01) in frozen–thawed sperm as compared to fresh sperm (Figure 2A). Similarly, significantly reduced expression (*p* < 0.01) of piR-121380 and PTPN7 was observed in capacitated sperm as compared to fresh sperm. Given that cryopreservation induces the degradation of mRNA, we further performed mRNA stability assay to explore the effect of cryopreservation on the mRNA level of PTPN7. As shown in Supplementary Figure S1, no significant differences were observed in PTPN7 mRNA half-life between fresh and frozen–thawed sperm (frozen–thawed versus fresh: 1.165 versus 1.288 h), illustrating that the changes in PTPN7 mRNA level were not caused by mRNA degradation during cryopreservation. Additionally, the mRNA expression of ERK1 and ERK2 was also shown as a supplement. The expression levels of ERK1 and ERK2 in frozen–thawed sperm were remarkably higher than those in fresh sperm; however, they were significantly decreased in the capacitated group compared to those in the NC group (Figure 2B).

### piR-121380 has a Facilitating Effect on Sperm Motility

**FIGURE 3:**
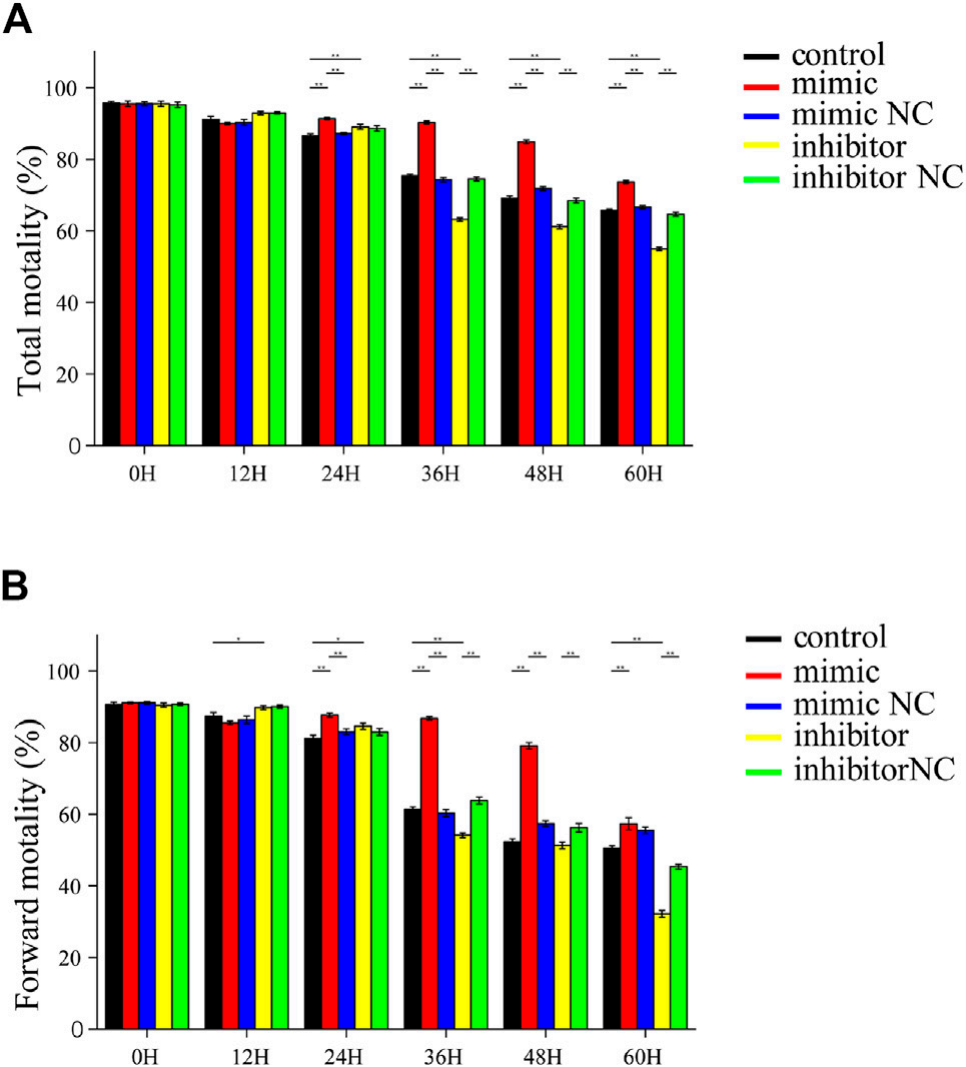
Sperm motility and forward motility in piR-121380 transfected sperm at 17°C. **(A)** Total sperm motility after transfection. **(B)** Sperm forward motility after transfection. The data are representative of at least three independent experiments (mean ± SEM).

Sperm total and forward motility, and kinetic parameters were measured using the CASA system after transfection of piR-121380 under liquid storage conditions (Figure 3A). Sperm motility was approximately 90% at 12 h in each group, and no significant differences were found among the other treatment groups. Sperm motility in the mimic group was significantly higher (*p* < 0.01) than that in the mimic NC group from 24 to 60 h. Conversely, sperm motility in the inhibitor group was significantly lower (*p* < 0.01) than that in the inhibitor NC group between 36 and 60 h (Figure 3B). Moreover, sperm forward motility showed synchronous changes with sperm motility except at 24 h. The highest sperm forward motility in the inhibitor group was consistent with the change of PTPN7 mRNA (Figure 1C). The kinetic parameters of piR-121380 transfected sperm are shown in Supplementary Table S1.

### Effect of piR-121380 on Sperm Acrosome Reaction

**FIGURE 4:**
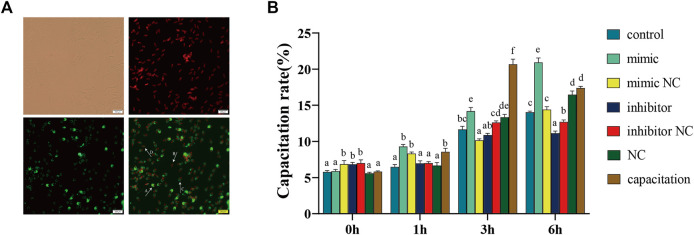
**(A)** Assessment of the acrosomal status and capacitation rate at 37°C by a PNA/PI staining assay. Furthermore, 40 ng/ml heparin was used to stimulate acrosome reaction as the positive control. A: integral acrosome (green, PI^−^/PNA^+^), B: dead sperm (red, PI^+^/PNA^−^), C: acrosomal reaction (green/red, PI^+^/PNA^+^), D: damaged acrosome and incomplete membrane (green/red, PI^+^/PNA^+^). **(B)** The capacitation rate is equal to the sum of C group divided by the total sperm. The data are representative of at least three independent experiments (mean ± SEM).

The PNA staining patterns correspond to different acrosomal statuses, which are shown in Figure 4A. The results showed that the capacitation rate of boar sperm in the mimic group was significantly higher than that in the mimic NC group, while the capacitation rate in the inhibitor group was significantly lower than that in inhibitor NC group at 3 and 6 h (Figure 4B). However, no differences were observed between the mimic or inhibitor group and their respective controls at 1 h. Compared to the capacitation group, the capacitation rate of the mimic group was continuously lower within 3 h.

### Effect of piR-121380 on Intracellular Ca2+ Concentration ([Ca2+]i) in Boar Sperm

**FIGURE 5:**
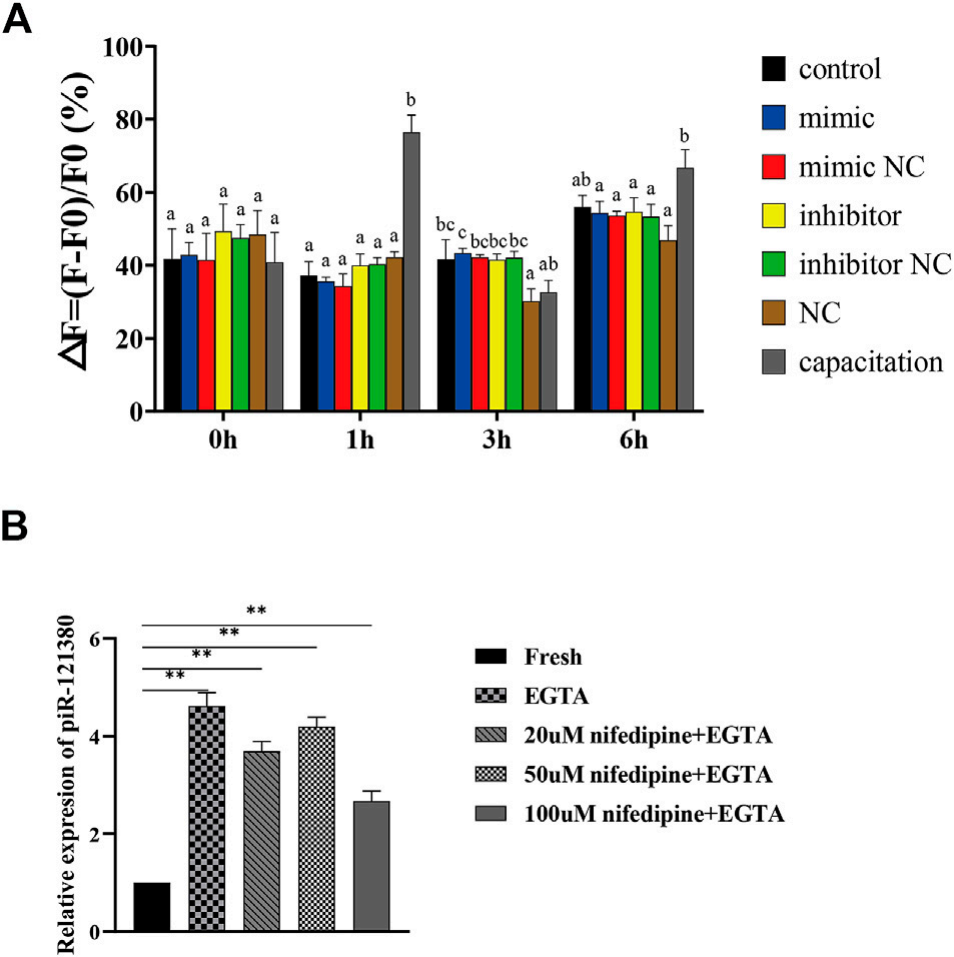
Effect of piR-121380 on [Ca^2+^]_i_ of boar sperm at 37°C. **(A)** After transfection and incubation with heparin, sperm were stained with 4 μM Fluo 4, am. The [Ca^2+^]_i_ was determined by the following equation: ΔF/F0 (%) = (F − F0)/F0 × 100%. F indicates the fluorescent intensity of each group, and F0 indicates the fluorescence intensity of sperm suspension without treatment. **(B)** The expression of piR-121380 after adding 5 μM EGTA, 20 μM nifedipine and 50 μM EGTA, 50 μM nifedipine and 50 μM EGTA, and 100 μM nifedipine and 50 μM EGTA, respectively, into sperm to inhibit the influx of Ca^2+^. The data are representative of at least three independent experiments (mean ± SEM).

Our data demonstrated that piR-121380 exhibited a facilitating effect on the intracellular Ca^2+^ concentration ([Ca2+]i)-dependent sperm functions, including sperm motility and capacitation rate, implying that piR-121380 may affect sperm Ca^2+^ signaling. Therefore, we further investigated whether piR-121380 affects boar sperm [Ca2+]i. Since the change of calcium ions is fast, we detected the intracellular concentration of Ca^2+^ at 0, 1, 3 and 6 h after transfection (Figure 5A). The results indicated that there were no statistically significant differences between the control group and transfection groups within 6 h. However, the sperm [Ca2+]i in the capacitation group was significantly higher than that in the control group at 1 and 6 h. To further identify the relationship between piR-121380 and intracellular calcium, we examined the relative expression of piR-121380 in calcium free medium. The results showed that the expression of piR-121380 was significantly increased (*p* < 0.01) when there was no extracellular Ca^2+^ (Figure 5B).

### Localization of PTPN7, ERK1, and ERK2 Proteins in Boar Sperm

**FIGURE 6:**
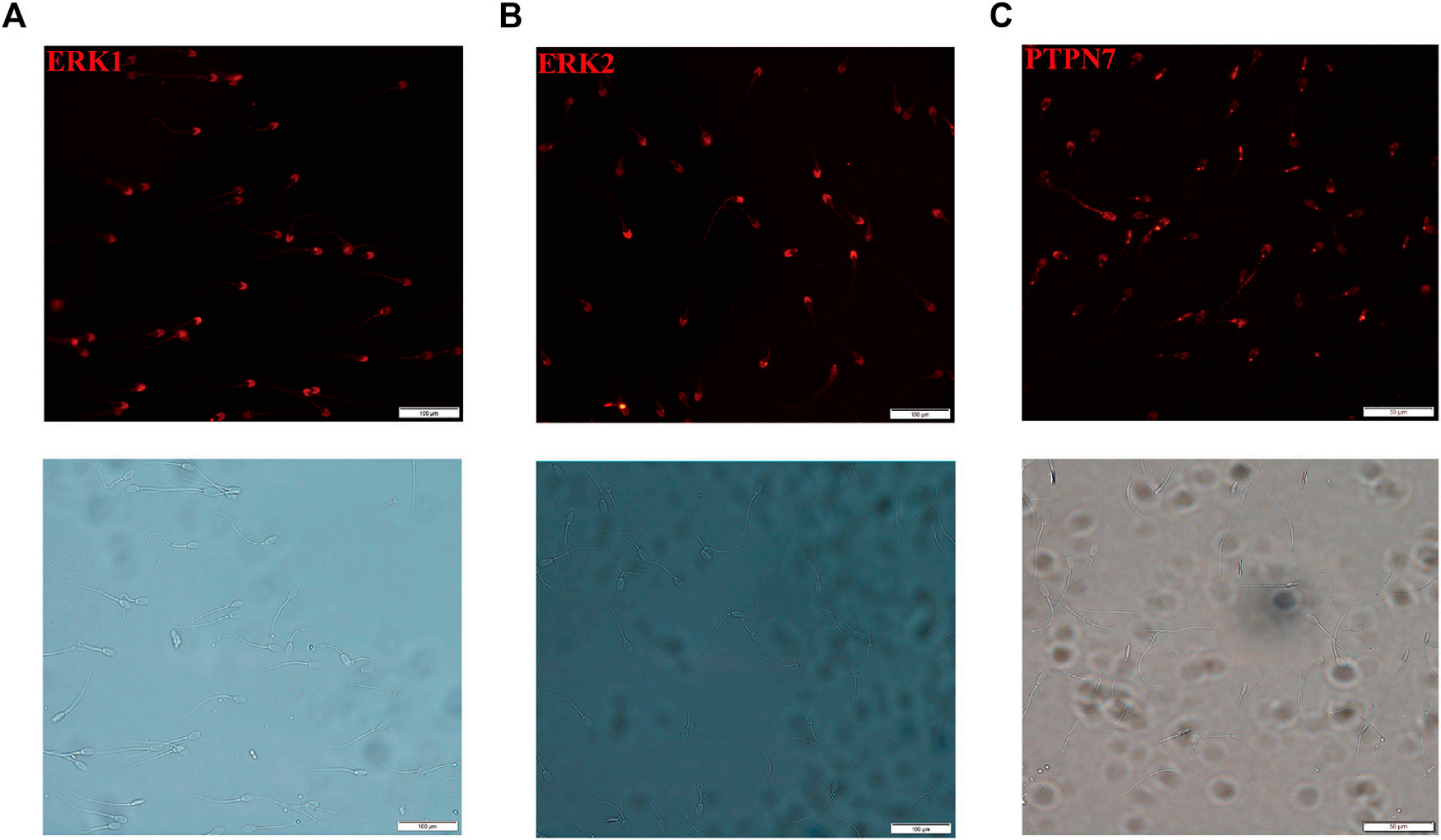
Localization of ERK1/2 and PTPN7 on boar sperm. **(A)** ERK1, **(B)** ERK2, and **(C)** PTPN7.

To clarify the localization of PTPN7, ERK1, and ERK2 in boar sperm, sperm without methanol treatment were incubated with anti-PTPN7, ERK1, and ERK2 antibodies. ERK1/2 was located on the head and distributed along the tail of mature ejaculated boar spermatozoa (Figures 6A,B). PTPN7 was mainly located on the head of boar sperm with a little distribution on the neck and tail (Figure 6C).

### piR-121380 Negatively Regulates ERK2 Phosphorylation by Targeting PTPN7

**FIGURE 7:**
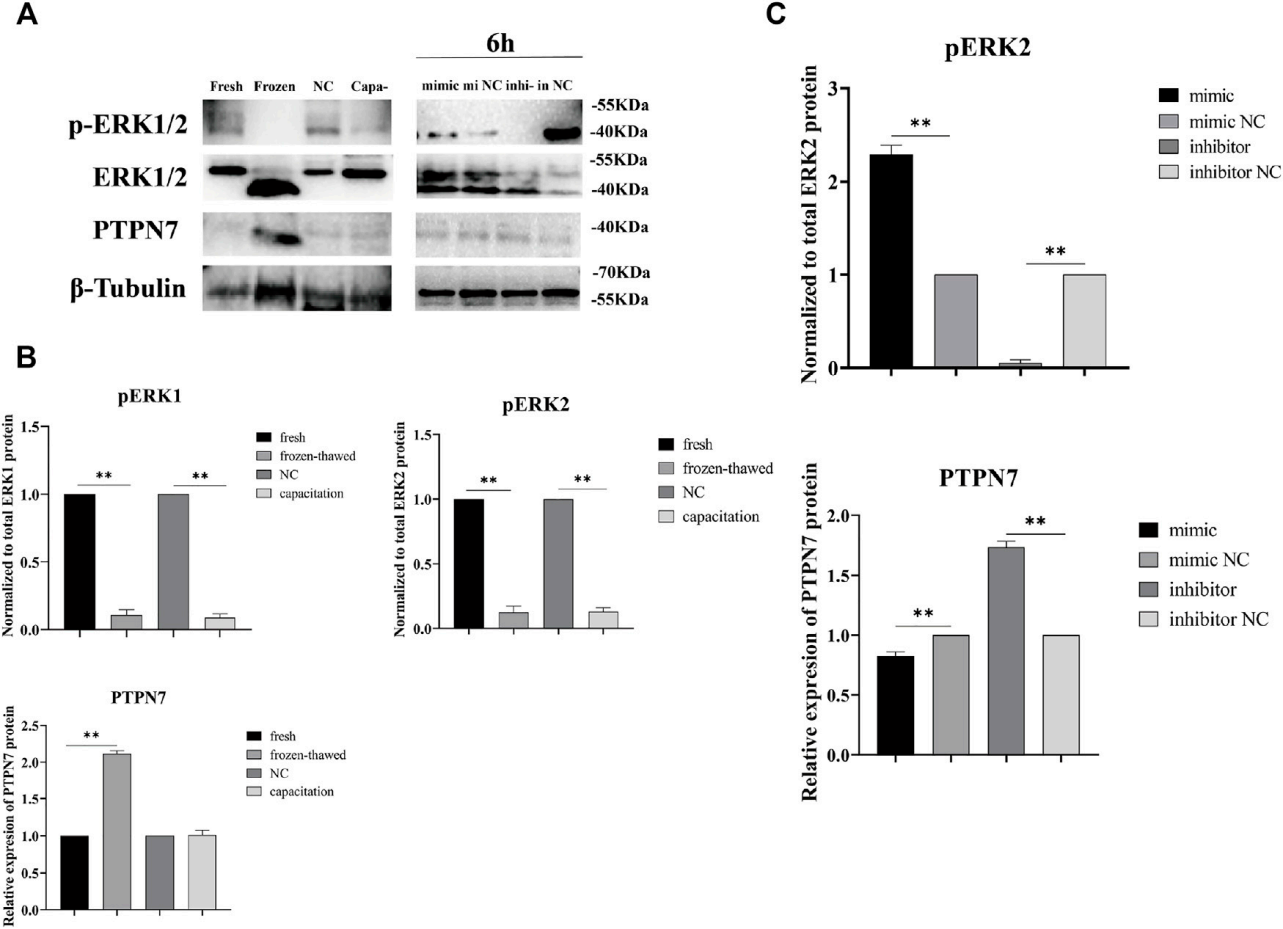
The protein levels of PTPN7, total ERK1/2, and phosphorylated ERK1/2 in boar sperm were measured by Western blot. **(A)** Anti-phosphorylated proteins were extracted from transfected, frozen–thawed, and capacitated sperm. Western blots performed using anti-ERK1/2, phospho-ERK1/2, PTPN7, and β-Tubulin antibodies. The results are representative of three independent experiments. **(B)** The protein levels of phosphorylated ERK1/2 and PTPN7 in fresh, frozen–thawed, NC, and capacitated sperm. “NC” means negative control group incubated at 37°C. “Capa-” means capacitation group. The protein level of phosphorylated ERK1/2 was quantified by normalization to total ERK1/2 protein using ImageJ, and the level of PTPN7 was quantified by normalization to β-Tubulin. **(C)** The protein levels of phosphorylated ERK2 and PTPN7 were shown in transfected sperm at 6 h.

To observe the effect of piR-121380 on ERK1/2 phosphorylation, and the difference in pattern of PTPN7 and ERK1/2 phosphorylation between cryopreservation and capacitation, we detected the protein levels of PTPN7, ERK1/2, and phosphorylated ERK1/2 in transfected, frozen–thawed, and capacitated sperm. Results showed that the protein level of PTPN7 was significantly decreased (*p* < 0.01) in the mimic group as compared to the mimic NC group at 6 h (Figure 7C). Conversely, the protein level of PTPN7 was significantly increased (*p* < 0.01) in the inhibitor group as compared to the inhibitor NC group at 6 h. Similarly, the protein level of PTPN7 was significantly increased (*p* < 0.01) in frozen–thawed sperm, which showed no significant difference between the NC group and capacitation group (Figure 7B). The protein level of phosphorylated ERK1/2, when normalized to total ERK1/2 protein, was significantly decreased (*p* < 0.01) in frozen–thawed and capacitation groups (Figure 7B). Interestingly, we found the existence of total ERK1/2 protein and phosphorylated ERK2 protein, without phosphorylated ERK1 protein at 6 h (Figure 7A). Results also showed that the protein level of phosphorylated ERK2, when normalized to total ERK2 protein, was significantly increased (*p* < 0.01) and decreased (*p* < 0.01) in the mimic group and inhibitor group at 6 h, respectively. The protein bands of phosphorylated ERK1/2 and PTPN7 in transfected sperm at 0 and 3 h are shown in Supplementary Figure S2.

## Discussion

In addition to the common changes during cryopreservation, the freezing–thawing procedure also affects other important components related to sperm functions, such as DNA integrity, messenger RNAs (mRNAs), and non-coding RNAs (Ran et al., 2019; Peris-Frau et al., 2021; Wang et al., 2021). Although significant progress has been made in the identification of markers of cryopreservation, including certain mRNA, miRNA transcripts, and proteins, little attention has been paid to piRNAs involved in cryoresistance. Our current study provides a novel insight that piR-121380 regulates boar sperm motility and is involved in sperm cryo-capacitation through phosphorylation of ERK2 by targeting PTPN7. We observed that PTPN7 was positively targeted by piR-121380 (Figure 1A). Following transfection with piR-121380 mimic and inhibitor, the mRNA expression of PTPN7, sperm motility and forward motility was increased and decreased, respectively (Figures 2A, 3), consistent with a previous study showing that sperm motilities of human and mouse were decreased with PTPN7 inhibition (Tomes et al., 2004). A previous study also explained the relationship between piRNAs and sperm motility (Capra et al., 2017). Furthermore, extracellular signal-regulated kinase (ERK) module of the MAPK pathway seems to promote sperm hyperactivated motility and acrosomal reaction (Almog et al., 2008; Li et al., 2009). In the present study, an increased phosphorylation of ERK2 was observed in the mimic group, suggesting that piR-121380 regulates sperm motility through phosphorylation of ERK2 by targeting PTPN7. Our findings provide molecular evidence and reveal a potential link between piR-121380, PTPN7, and sperm motility.

Although the central role of cAMP/PKA/protein tyrosine phosphorylation cascade has been identified in cryo-capacitation changes, the molecular mechanism of protein tyrosine phosphorylation during cryopreservation has not been fully investigated. Multiple subtypes of PTP have been identified in mouse, human, and pig sperm (except for PTPN7), all of which are involved in sperm motility and capacitation (Tomes et al., 2004; Gonzalez-Fernandez et al., 2009). In this study, we first reported the pattern of PTPN7 protein in boar sperm, falling into class I cysteine-based PTPs. Compared to fresh sperm, the protein level of PTPN7 was significantly increased (*p* < 0.01) in frozen–thawed sperm, but no differences were observed between fresh and capacitated sperm (Figure 7B), implying the different patterns of PTPN7 between cryo-capacitation and true capacitation. To further investigate whether cryopreservation affects ERK1/2 phosphorylation *via* PTPN7, we showed that after transfection with piR-121380 inhibitor, the levels of phosphorylated PTPN7 and ERK2 were increased and decreased, respectively (Figures 7B,C), consistent with the negative regulation relationship between PTPN7 and ERK2 identified in somatic cells reported previously (Inamdar et al., 2019). Normally, protein tyrosine phosphorylation occurs in the head of sperm, such as a kinase anchor protein (AKAP82), fibrous sheath protein of 95 kDa (FSP95), and calcium-binding tyrosine phosphorylation-regulated protein (CABYR), all of which play an important role in hyperactivation (Johnson et al., 1997; Mandal et al., 1999; Naaby-Hansen et al., 2002). In this study, the distribution of PTPN7 overlapped with that of ERK1/2 in acrosomal compartments, which helps elucidate the involvement of PTPN7 and ERK2 phosphorylation in boar sperm motility.

Notably, after transfection with piR-121380 mimic, the decreased mRNA expression of PTPN7 was inconsistent with the amount of PTPN7 protein, which was similar with the changes during cryopreservation (Figures 2A, 7B). Comparing PTPN7 mRNA half-life between fresh and frozen–thawed sperm (Supplementary Figure S1) leads us to believe that decreased PTPN7 mRNA expression induced by cryopreservation results in PTPN7 protein synthesis and is not a product of mRNA degradation. Although transcription and translation are considered silent events in spermatozoa, there are many mRNA transcripts that can result in promotion of translation for protein synthesis (Lv et al., 2020; Lymbery et al., 2020).

Phosphorylation of ERK1/2 has been demonstrated to be tightly coupled with intracellular calcium concentration (Jaldety and Breitbart, 2015). Influx of Ca^2+^ is one of the decisive events to regulate sperm motility, acrosomal reaction, and chemotaxis (Trevino et al., 2006; Mizuno et al., 2012; Liu et al., 2018). The influx of Ca^2+^ is mainly carried by the CatSper channels located on sperm flagellar. It is well known that cryopreservation impairs the CatSper channel due to the loss of cholesterol, resulting in a massive influx of Ca^2+^ into the sperm, further leading to activation of a set of soluble adenylate cyclase (sAC)/cAMP/PKA (Gungor et al., 2021). In this study, no significant differences in the concentration of intracellular calcium were observed after transfection with piR-121380 mimic and inhibitor. However, the expression of piR-121380 was significantly increased (*p* < 0.01) in the absence of extracellular Ca^2+^ in the culture medium. Therefore, we believe that piR-121380, downstream of calcium influx, regulates PTPN7 to affect sperm motility.

In addition to the roles in sperm motility, piR-121380 also plays an important role in acrosomal reaction in sperm. In our study, increased and decreased capacitation rates were observed in mimic and inhibitor groups, respectively. Defective capacitation and failure to bind and penetrate the zona pellucida have been reported in pachytene piRNA mutant sperm (Wu et al., 2020). Choi et al. found sperm acrosome growth and infertility in mice lacking chromosome 18 pachytene piRNA (Choi et al., 2021). In light of the significant regulatory role of ERK1/2 in boar sperm capacitation (Awda and Buhr, 2010), piR-121380 might have an indirect role in regulating acrosomal reaction through ERK2 phosphorylation in mature boar sperm.

## Conclusion

In this study, we demonstrated that the mRNA expression of piR-121380 was significantly decreased in post-thawed boar sperm. Further studies showed that piR-121380 regulates boar sperm motility and is involved in sperm cryo-capacitation through phosphorylation of ERK2 *via* targeting PTPN7. We explored the role of piRNA in the process of sperm cryo-capacitation, which helps to further understand the molecular mechanism of cryo-capacitation and improve the quality of frozen–thawed boar sperm.

## Data Availability

The original contributions presented in the study are included in the article/Supplementary Material, further inquiries can be directed to the corresponding author.
