# Supplementary material for: piR-121380 Is Involved in Cryo-Capacitation and Regulates Post-Thawed Boar Sperm Quality Through Phosphorylation of ERK2 via Targeting PTPN7
Source: Front Cell Dev Biol. 2022 Jan 26;9:792994. doi: 10.3389/fcell.2021.792994 (PMC8826432; doi:10.3389/fcell.2021.792994)
Supplement: Supplementary file 1 [file Table1.DOCX]

## Table S1 The sperm kinetic parameters from 12 to 60h after piR-121380 transfection.

|  | Time (h) | Groups | | | | |
| --- | --- | --- | --- | --- | --- | --- |
|  |  | Control | mimic | mimic NC | inhibitor | inhibitor NC |
| VCL | 0 | 121.71±0.46^a^ | 121.66±0.47^a^ | 121.83±0.37^a^ | 121.38±0.40^a^ | 121.95 ±0.47^a^ |
|  | 12 | 96.85±2.10^c^ | 88.71±2.19^b^ | 80.52±2.29^a^ | 81.67±1.34^a^ | 96.64±1.16^c^ |
|  | 24 | 76.36±2.67^a^ | 85.35±2.38^b^ | 81.89±3.64^ab^ | 75.94±2.47^a^ | 80.98±3.36^ab^ |
|  | 36 | 47.13±2.09^a^ | 91.23±1.31^c^ | 55.64±2.05^b^ | 55.16±1.30^b^ | 55.56±1.48^b^ |
|  | 48 | 41.14±2.01^a^ | 49.86±0.89^b^ | 51.43±1.71^b^ | 52.56±1.98^b^ | 49.57±1.60^b^ |
| VSL | 60 | 42.23±0.61^b^ | 49.19±2.51^c^ | 49.46±1.36^c^ | 34.22±0.68^a^ | 45.00±0.70 ^b^ |
|  | 0 | 35.28±0.26^a^ | 35.16±0.17^a^ | 35.48±0.26^a^ | 35.16±0.22^a^ | 35.46±0.34 ^a^ |
|  | 12 | 30.89±0.90^b^ | 26.71±0.77^a^ | 25.37±1.26^a^ | 24.50±0.52^a^ | 29.70±0.51^b^ |
|  | 24 | 22.26±0.78^a^ | 24.55±1.02^a^ | 23.30±1.66^a^ | 23.24±0.77^a^ | 24.24±1.15^a^ |
| VAP | 36 | 17.84±0.58^a^ | 25.72±0.48^c^ | 20.79±1.12^b^ | 24.32±0.77^c^ | 21.51±1.00^b^ |
|  | 48 | 13.63±0.48^a^ | 14.38±0.52^a^ | 18.92±0.95^b^ | 23.39±0.97^c^ | 20.13±0.97^b^ |
|  | 60 | 17.87±0.51^a^ | 19.56±0.98^ab^ | 20.95±0.87^bc^ | 17.63±0.58^a^ | 22.95±0.78^c^ |
|  | 0 | 59.72±0.37^a^ | 58.90±0.27^a^ | 59.34±0.36^a^ | 59.94±0.25^a^ | 59.69±0.39^a^ |
|  | 12 | 44.59±1.01^c^ | 39.58±0.90^b^ | 39.44±1.20^b^ | 36.63±0.70^a^ | 43.86±0.60^c^ |
|  | 24 | 33.84±1.14^a^ | 37.56±1.40^a^ | 36.11±2.02^a^ | 35.29±0.94^a^ | 37.89±1.32^a^ |
| BCF | 36 | 27.22±0.75^a^ | 39.29±0.55^d^ | 32.38±1.37^bc^ | 34.90±0.87^c^ | 30.36±1.02^b^ |
|  | 48 | 21.58±0.44^a^ | 22.05±0.56^a^ | 29.65±1.16^b^ | 29.46±1.59^b^ | 29.01±0.92^b^ |
|  | 60 | 25.16±0.53^b^ | 28.49±1.11^c^ | 30.21±1.12^cd^ | 21.90±0.58^a^ | 31.25±0.78^d^ |
|  | 0 | 11.93±0.21^a^ | 11.98±0.41^a^ | 12.02±0.49^a^ | 12.06±0.34^a^ | 12.11±0.28^a^ |
|  | 12 | 11.00±0.28^c^ | 10.35±0.26^b^ | 8.64±0.14^a^ | 10.05±0.17^b^ | 11.26±0.14^c^ |
| STR | 24 | 8.40±0.36^a^ | 9.75±0.38^b^ | 9.41±0.41^ab^ | 8.47±0.25^a^ | 8.94±0.30^ab^ |
|  | 36 | 4.99±0.23^a^ | 9.23±0.16^c^ | 5.54±0.09^a^ | 5.12±0.08^a^ | 6.22±0.18^b^ |
|  | 48 | 4.47±0.07^a^ | 5.73±0.10^d^ | 5.30±0.09^c^ | 4.91±0.14^b^ | 5.46±0.12^cd^ |
|  | 60 | 4.09±0.05^b^ | 5.63±0.35^c^ | 5.26±0.12^c^ | 2.97±0.15^a^ | 3.62±0.08^b^ |
|  | 0 | 0.59±0.01^a^ | 0.60±0.01^a^ | 0.60±0.01^a^ | 0.59±0.01^a^ | 0.59±0.01^a^ |
|  | 12 | 0.69±0.02^b^ | 0.67±0.01^ab^ | 0.65±0.03^a^ | 0.67±0.00^ab^ | 0.68±0.00^ab^ |
|  | 24 | 0.66±0.01^a^ | 0.65±0.01^a^ | 0.64±0.02^a^ | 0.66±0.01^a^ | 0.64±0.01^a^ |
|  | 36 | 0.65±0.01^a^ | 0.65±0.00^a^ | 0.63±0.01^a^ | 0.69±0.01^b^ | 0.71±0.02^b^ |
|  | 48 | 0.63±0.01^a^ | 0.64±0.01^ab^ | 0.63±0.01^a^ | 0.81±0.04^c^ | 0.69±0.01^b^ |
|  | 60 | 0.71±0.01^b^ | 0.68±0.01^a^ | 0.69±0.00^ab^ | 0.80±0.01^d^ | 0.73±0.01^c^ |
| LIN | 0 | 0.29±0.00^a^ | 0.29±0.00^a^ | 0.29±0.00^a^ | 0.29±0.00^a^ | 0.29±0.00^a^ |
|  | 12 | 0.32±0.01^a^ | 0.30±0.01^a^ | 0.31±0.01^a^ | 0.30±0.00^a^ | 0.31±0.00^a^ |
|  | 24 | 0.29±0.01^ab^ | 0.29±0.01^ab^ | 0.28±0.01^a^ | 0.31±0.01^b^ | 0.30±0.01^ab^ |
|  | 36 | 0.38±0.01^b^ | 0.28±0.00^a^ | 0.37±0.01^b^ | 0.44±0.01^c^ | 0.39±0.01^b^ |
|  | 48 | 0.34±0.02^b^ | 0.29±0.01^a^ | 0.36±0.01^b^ | 0.44±0.01^d^ | 0.40±0.01^c^ |
|  | 60 | 0.42±0.01^a^ | 0.40±0.01^a^ | 0.42±0.01^a^ | 0.51±0.01^b^ | 0.51±0.01^b^ |
| WOB | 0 | 0.49±0.00^a^ | 0.48±0.00^a^ | 0.49±0.00^a^ | 0.49±0.00^a^ | 0.49±0.00^a^ |
|  | 12 | 0.46±0.01^a^ | 0.45±0.01^a^ | 0.49±0.02^b^ | 0.45±0.00^a^ | 0.45±0.00^a^ |
|  | 24 | 0.44±0.01^ab^ | 0.43±0.00^a^ | 0.44±0.01^ab^ | 0.47±0.01^ab^ | 0.47±0.01^b^ |
|  | 36 | 0.58±0.01^c^ | 0.43±0.00^a^ | 0.58±0.01^c^ | 0.63±0.00^d^ | 0.55±0.01^b^ |
|  | 48 | 0.53±0.02^b^ | 0.44±0.01^a^ | 0.57±0.00^c^ | 0.56±0.02^bc^ | 0.59±0.01^c^ |
|  | 60 | 0.59±0.01^a^ | 0.59±0.01^a^ | 0.61±0.01^a^ | 0.64±0.01^b^ | 0.69±0.01^c^ |
| ALH | 0 | 1.25±0.02^a^ | 1.24±0.02^a^ | 1.23±0.01^a^ | 1.27±0.01^a^ | 1.24±0.01^a^ |
|  | 12 | 1.22±0.02^c^ | 1.10±0.03^b^ | 1.00±0.02^a^ | 1.06±0.01^b^ | 1.17±0.01^c^ |
|  | 24 | 1.04±0.03^a^ | 1.17±0.03^b^ | 1.12±0.02^ab^ | 1.05±0.03^a^ | 1.13±0.04^ab^ |
|  | 36 | 0.61±0.02^a^ | 1.19±0.01^c^ | 0.67±0.02^b^ | 0.60±0.01^a^ | 0.69±0.01^b^ |
|  | 48 | 0.51±0.01^a^ | 0.71±0.01^d^ | 0.64±0.01^c^ | 0.57±0.02^b^ | 0.61±0.01^bc^ |
|  | 60 | 0.52±0.00^c^ | 0.62±0.03^d^ | 0.59±0.01^d^ | 0.36±0.01^a^ | 0.47±0.01^b^ |

Note: Differences among groups were analyzed by one-way analysis of variance (ANOVA) followed by the Dunnett post-test. Different superscripts in the same row of data represent significant differences (P < 0.05), and the same superscripts represent non-significant differences (P > 0.05). VCL: The speed of curve movement, VSL: The speed of linear movement, VAP: The speed of average path, BCF: Whipping frequency, LIN: Linearity, namely VSL/VCL, STR: Straight, namely VSL/VAP, WOB: namely VAP/VCL, ALH: Amplitude of lateral head displacement.
