# Supplementary figures and images for: piR-121380 Is Involved in Cryo-Capacitation and Regulates Post-Thawed Boar Sperm Quality Through Phosphorylation of ERK2 via Targeting PTPN7
Source: Front Cell Dev Biol. 2022 Jan 26;9:792994. doi: 10.3389/fcell.2021.792994 (PMC8826432; doi:10.3389/fcell.2021.792994)

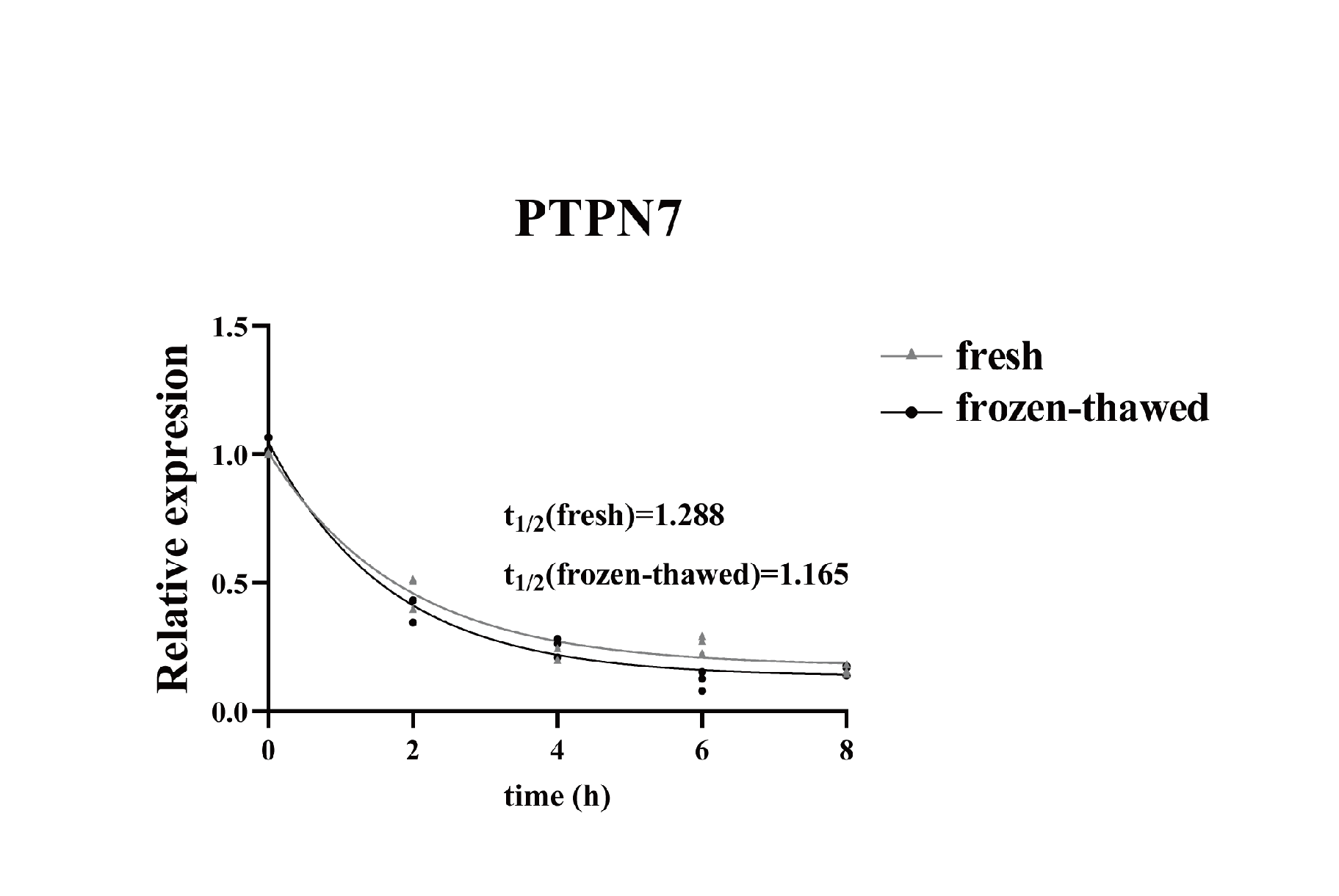

Supplement: Supplementary file 2 [file Image1.JPEG]

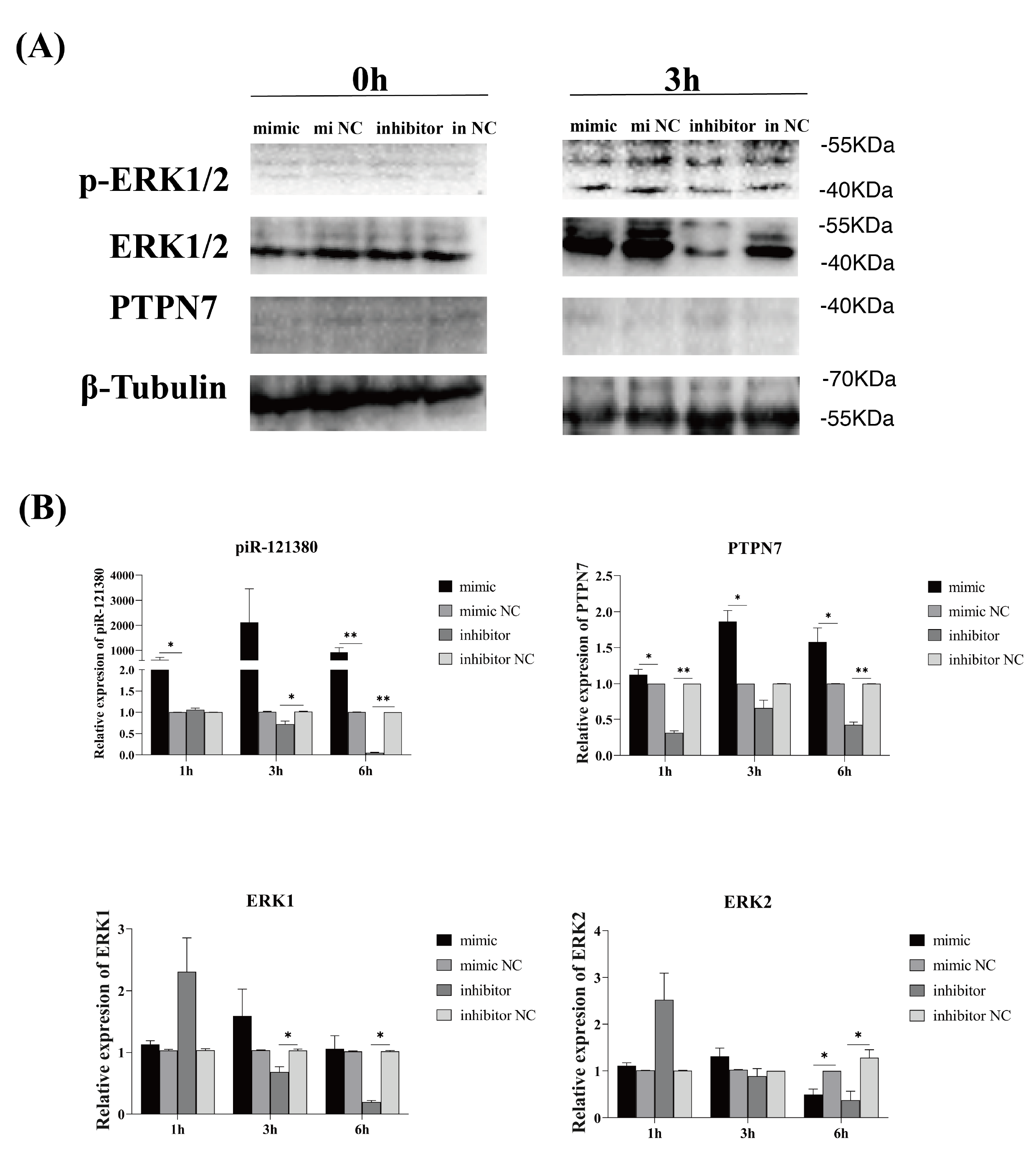

Supplement: Supplementary file 3 [file Image2.JPEG]
