# Supplementary material for: piR-121380 Is Involved in Cryo-Capacitation and Regulates Post-Thawed Boar Sperm Quality Through Phosphorylation of ERK2 via Targeting PTPN7
Source: Front Cell Dev Biol. 2022 Jan 26;9:792994. doi: 10.3389/fcell.2021.792994 (PMC8826432; doi:10.3389/fcell.2021.792994)
Supplement: Supplementary file 4 [file Table2.DOCX]

Raw data accessibility

1. FIGURE1 Dual Luciferase Reporter Assay: <https://www.jianguoyun.com/p/DRdRlNQQ5eD2CRje_5QE>
2. FIGURE2 qPCR: <https://www.jianguoyun.com/p/DcWD_coQ5eD2CRjk_5QE>
3. FIGURE3 sperm motility: <https://www.jianguoyun.com/p/DZTnW1cQ5eD2CRjm_5QE>
4. FIGURE4 Ca^2+^: <https://www.jianguoyun.com/p/DekE_IEQ5eD2CRjq_5QE>
5. FIGURE5 Capacitation rate: <https://www.jianguoyun.com/p/DTA1uh8Q5eD2CRjs_5QE>
6. FIGURE6 Immunofluorescence: <https://www.jianguoyun.com/p/DSJjRD0Q5eD2CRju_5QE>
7. FIGURE7 Western Blot: <https://www.jianguoyun.com/p/DR9oGqQQ5eD2CRjw_5QE>
8. Supplementary Table1 sperm parameters: <https://www.jianguoyun.com/p/DTiNwPkQ5eD2CRj1_5QE>
9. Supplementary Figure1: <https://www.jianguoyun.com/p/DVR5s2EQ5eD2CRj5hKIE>
10. Supplementary Figure2: <https://www.jianguoyun.com/p/DcBoKeQQ5eD2CRjy_5QE>
11. Sperm Viability:

<https://www.jianguoyun.com/p/DQZ-q3YQ5eD2CRjKg6IE>
